# Refining the radiation and contrast medium dose in weight‐grouped scanning protocols for coronary CT angiography

**DOI:** 10.1002/acm2.14041

**Published:** 2023-05-21

**Authors:** Yan Zhang, Ying Wang, Jing Li, Guozhi Zhang, Aihui Di, Huishu Yuan

**Affiliations:** ^1^ Department of Radiology Peking University Third Hospital Beijing China; ^2^ United Imaging Healthcare Shanghai China

**Keywords:** coronary computed tomography angiography, image quality, iodine delivery rate, radiation dose

## Abstract

**Purpose:**

To refine the currently used, weight‐grouped protocol for coronary computed tomography angiography (CCTA), in terms of the radiation and contrast medium dose, through clinical evaluation.

**Methods:**

Following the current routine setting that varies between three weight groups (group A: 55–65 kg, group B: 66–75 kg, group C: 76–85 kg), three additional reduction protocols were proposed to each group, with different combinations of lowered tube voltage (70–100 kVp), tube current (100–220 mAs), and iodine delivery rate (0.8–1.5 gI/s). A total of 321 patients scheduled for CCTA due to suspected coronary artery disease were enrolled, who were randomly assigned to one of the four subgroups of settings under the corresponding weight group. The resulting objective image quality was compared by measuring the contrast‐to‐noise ratio and signal‐to‐noise ratio. Subjective image quality was graded by two radiologists using a 4‐point Likert scale, on a total of 3848 segments. The optimal protocol for each weight group was determined with respect to the image quality and the applied radiation dose.

**Results:**

For all three groups, no significant difference was noticed in objective images quality between subgroups of dose settings (all *p* > 0.05). The average score on subjective image quality was ≥3 for every subgroup, while the percentage of score 4 showed greater dependence on the setting, ranging from 83.2% to 91.5%, and was chosen to be the determining factor. The optimal dose settings were found to be 80 kVp, 150 mAs, and 1.0 gI/s for patients of 55–75 kg in weight, and 100 kVp, 170 mAs, and 1.5 gI/s for those of 76–85 kg.

**Conclusion:**

It is feasible to refine the currently used, weight‐grouped protocol for CCTA in terms of radiation and contrast medium dose, by use of an optimization strategy where the balance between dose and image quality can be improved in a routine clinical setting.

## 1 INTRODUCTION

Coronary computed tomography angiography (CCTA) is a non‐invasive imaging modality that is of great value for diagnosing coronary artery disease (CAD).^1^, ^2^ As one of the first examinations for patients suspected of CAD, CCTA is crucial to clinical decision making, which makes it rather important to ensure a diagnostically sufficient CCTA image quality.^3^ Motion artifacts and vessel enhancement are two major factors relating to the image quality of CCTA in practice.^4^ Tackling with motion artifacts is in fact a process of optimizing the way of CT acquisition in regard to the cardiac cycle.^5^, ^6^, ^7^ While applying extra heart rate control via medication and implementing the so‐called retrospective acquisition mode are both efficient measures, the use of a high‐speed wide‐detector CT scanner certainly has its advantage for the ability of acquiring all necessary data within one single heartbeat and less than one rotation.^8^, ^9^, ^10^ In addition to the hardware, software solutions by means of motion correction in image post processing have also become a common choice, although the principle of various correction algorithms as well as the scanners may both largely differ.^11^, ^12^, ^13^

Efforts have also been made in optimizing the vessel enhancement in order to improve the image quality of CCTA.^14^, ^15^, ^16^ Mihl et al. evaluated a body weight adapted protocol for contrast injection, where a more homogeneous enhancement pattern was achieved between different weight groups while the volume of contrast media could be reduced for the majority of patients.^17^ Andreini et al. demonstrated the feasibility of using reduced iodine concentration and an 80 kVp tube voltage on patients with low body weight.^18^ Tan et al. proposed a formula for calculating the personalized volume of contrast medium at a high delivery rate for use in low tube voltage CCTA examination.^19^ Similar to results found in earlier studies, these investigations have made one central suggestion in common, which was to apply reduced dose of contrast media in CCTA with a lower tube voltage.^14^, ^16^ While this is particularly beneficial as proven to patients with relatively low body weight, it remains unclear whether the same can be achieved for an individual that falls in the general patient population, and if it is so, how the complete optimal setting would be. As such, a more systematic optimization strategy based on various scanning parameters and accounting for both radiation and contrast dose is worth investigation for practical implementation.

The purpose of this study was to refine the currently used, body weight grouped protocol for CCTA in terms of the radiation and contrast medium dose, hopefully reducing the dose while improving or at least not affecting the image quality. To do so in a routine clinical setting, the reduction was restricted within an empirical extent that would not severely compromise the image quality and leave the diagnosis totally impossible. The search for optimal protocol was subject to comprehensive comparison of the resulting subjective and objective image quality as well as the applied radiation dose.

## 2 METHODS

### 2.1 Study design and participants

**FIGURE 1:**
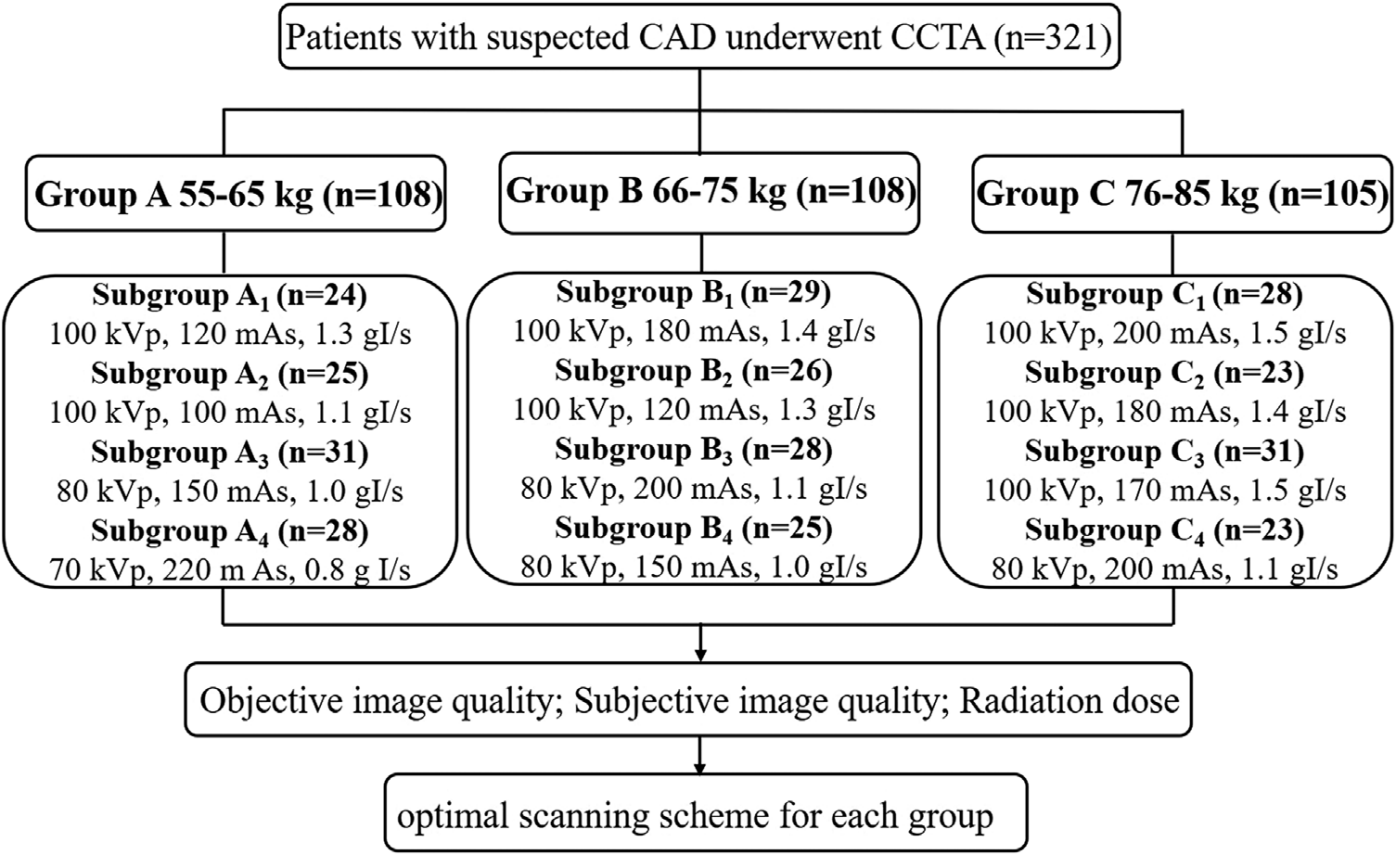
Flowchart of the study design. CAD, coronary artery disease; CCTA, coronary computed tomography angiography.

As illustrated in Figure 1, the refining strategy was designed to begin with lowering the radiation and iodine dose routinely used for each of the three body weight groups, that is, 55−65, 66−75, 76−85 kg. For each group, three ‘reduction’ protocols, in addition to the current one, were tested, applying different combinations of lowered tube voltage (70, 80, and 100 kVp), tube current (100−220 mAs), and iodine delivery rate (IDR, 0.8−1.5 gI/s). Specifically, subgroups A_2_‐A_4_ followed the settings of A_1_, and so forth. More details were listed in Table 1. It was worth mentioning that there were overlaps of the settings for test between adjacent weight groups.

**TABLE 1:** Radiation and Contrast medium dose parameters.

	Tube voltage (kVp)	Tube current (mAs)	Planned volume CT dose index (CTDI_vol_, mGy)	Iodine delivery rate (gI/s)	Volume of contrast media (mL)
Group A 55−65 kg					
Subgroup A_1_	100	120	12.78	1.3	43
Subgroup A_2_	100	100	10.65	1.1	38
Subgroup A_3_	80	150	7.33	1.0	35
Subgroup A_4_	70	220	6.26	0.8	27
Group B 66−75 kg					
Subgroup B_1_	100	180	19.17	1.4	48
Subgroup B_2_	100	120	12.78	1.3	43
Subgroup B_3_	80	200	9.77	1.1	38
Subgroup B_4_	80	150	7.33	1.0	35
Group C 76−85 kg					
Subgroup C_1_	100	200	22.19	1.5	53
Subgroup C_2_	100	180	19.17	1.4	48
Subgroup C_3_	100	170	18.10	1.5	53
Subgroup C_4_	80	200	9.77	1.1	38

This prospective study received ethical approval from the Institutional Ethics Committee. Patients scheduled for CCTA from February to December 2022 due to suspected or known CAD were enrolled in this study, who were first categorized by body weight and then randomly assigned to one of the corresponding subgroups. Patients with implanted coronary stents were excluded because metal artifacts may be involved, which is important but a separate issue beyond dose and vessel enhancement as considered in the present study. Aside from the current study design, investigation on patients < 55  or > 85 kg was additionally carried out with a small number of cases, where the consideration on optimization might be different. Written informed consent was obtained from each participant before the CT examination.

To test whether there was a potentially better setting than the current one, and if so, to determine which the optimal setting was for each group, the process of balancing as well as the priority placed on dose or image quality may vary from case to case. On image quality, it must be noted that the aim was not to evaluate if the resulting images were acceptable for clinical diagnosis but to investigate, with respect to those routinely accepted, the possibility and the associated cost for improvement, which raised the baseline for comparison.

### 2.2 CT image acquisition

All CT examinations were performed on a 320‐row CT scanner (uCT 960+; United Imaging Healthcare; Shanghai; China) with prospective electrocardiography (ECG)‐gating in axial scan mode, where the acquisition was completed in one single heartbeat. As in the majority of routine cases, beta‐blockers were not used. All patients were trained for breath‐holding.

The scan range covered the entire heart, from 1 cm below the carina up to the diaphragm, corresponding to a z‐coverage of 12 , 14 , or 16 cm depending on the patient's heart size. The gantry rotation time was 0.25 s. Contrast medium with an iodine concentration of 350 mg/mL (Ioversol, Bayer) was used for all patients with 10‐s injection time, followed by 40 mL of saline solution. Data acquisition was triggered using a bolus tracking technique, which the region of interest (ROI) was set on the descending aorta at 1 cm below the tracheal bifurcation, with a triggering threshold of 100 Hounsfield Units (HU) and 6 s delay time. The optimal cardiac phase was automatically selected based on the measured ECG signal (ePhase; United Imaging Healthcare; Shanghai; China).

After scan, an artificial intelligence (AI)‐based software algorithm (CardioCapture; United Imaging Healthcare; Shanghai; China) was used for motion correction.^12^ Next, all CT images were reconstructed with a routinely available hybrid iterative algorithm (Karl 3D; United Imaging Healthcare; Shanghai; China), with a 512 × 512 pixels matrix, 0.5 mm slice thickness, and 0.5 mm slice interval. The data were then transferred to the clinical post‐processing workstation (uWS‐CT: R005; United Imaging Healthcare; Shanghai; China) to generate volume rendering (VR), maximum intensity projection (MIP), and curved planar reconstruction (CPR) images for each of the following analysis.

### 2.3 Objective image quality

One radiologist with over 7 years of experience in cardiovascular radiology sketched the ROIs for evaluating the objective image quality. The mean CT number and the noise were measured on the proximal segment of the left anterior descending (LAD) artery, the distal segment of the right coronary artery (RCA), as well as the adjacent pericardial fat. The ROI size on the pericardial fat was 12 mm^2^. Other ROIs were made as large as possible according to the lumen of the vessel (1.2 –1.7 mm^2^), away from the wall and any possible calcification or plaque. The noise was defined as the standard deviation (SD) of CT number within the ROI. The signal‐to‐noise ratios (SNRs) and contrast‐to‐noise ratios (CNRs) were calculated using:

(1) $$\begin{array}{c} SNR\;=\mu_{\mathrm{artery}}/SD_{\mathrm{artery}}\;\# \end{array}$$

(2) $$CNR\;=\left(\mu_{\mathrm{artery}}-\mu_{\mathrm{fat}}\right)/SD_{\mathrm{fat}}\;\#$$

where *μ* stands for the mean of CT number within the ROI.

### 2.4 Subjective image quality

**FIGURE 2:**
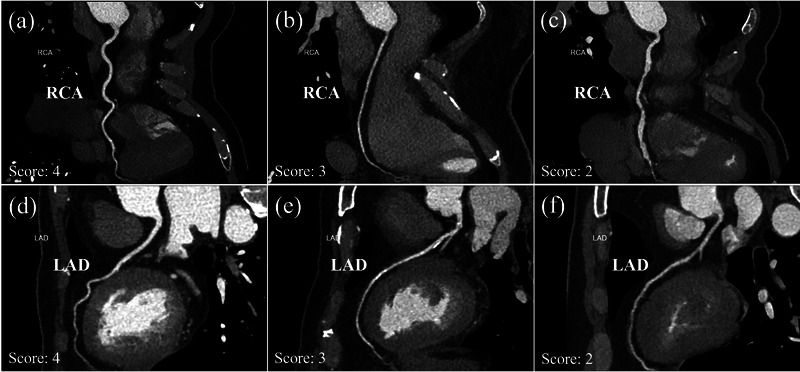
Coronary CT angiography: examples of different levels of image quality. Right coronary artery (RCA) with score of 2−4 (a‐c); Left anterior descending (LAD) coronary artery with score of 2−4 (d‐f).

Two radiologists, one with 5 years and the other with 10 years of experience in CCTA diagnosis, were asked to assess the subjective image quality independently, where the cases were randomized and the observers were kept blind to the scanning protocol. The evaluation was performed for vessels with a diameter > 1.5 mm on MIP reconstruction images according to the 18‐segment classification system.^20^ All segments were graded using a 4‐point Likert scale, accounting for contrast enhancement, vessel visualization, and noise appearance (score 4 = excellent; score 3 = acceptable; score 2 = inferior; score 1 = non‐acceptable).^6^ A score ≥ 3 was considered acceptable for diagnosis, as illustrated in Figure 2. In case of disagreement, consensus was obtained from two radiologists upon discussion.

### 2.5 Statistical analysis

Statistical analysis was performed using the SPSS software Version 26.0 (IBM Corp., Armonk, New York, USA). All data were expressed as means ± SD. The SNR, CNR, CTDI_vol_, and subjective scores of image quality were compared using the Kruskal−Wallis H test. The Kappa test was used to evaluate the interobserver variability (Kappa values): 0.81−1.0, excellent agreement; 0.61−0.80, good agreement; 0.41−0.60, moderate agreement; 0.21−0.40, inadequate agreement; less than 0.20, poor agreement). A *p* ≤ 0.05 was considered statistically significant.

## 3 RESULTS

### 3.1 Patient characteristics

A total of 321 patients (female/male: 126/195) were enrolled in the study, with 108, 108, and 105 cases in groups A, B, and C, respectively. Detailed information for each group i shown in Table 2. The age and heart rate did not differ significantly within four subgroups of each weight group (*p* > 0.05). Hypertension was found most common in all groups, with the highest percentage in group C. The stenosis severity and the presence of myocardial bridge both showed a trend across the three body weight groups.

**TABLE 2:** Patient characteristics for three weight groups.

Groups	Group A: 55−65 kg	Group B: 66−75 kg	Group C: 76−85 kg
Age (years)	62.29 ± 11.39	58.19 ± 11.39	58.53 ± 11.56
Gender (female/male)	108 (76/32)	108 (39/69)	105 (11/94)
Body mass index (BMI)	23.17 ± 4.28	25.24 ± 1.91	26.86 ± 1.88
Heart rate (bpm)	69 ± 13	66 ± 11	66 ± 12
Clinical history			
Hypertension	63 (58%)	66 (61%)	73 (70%)
Type II diabetes	25 (21%)	16 (15%)	23 (22%)
CAD family history	23 (20%)	20 (19%)	32 (30%)
Coronary dominance			
Right‐sided	90 (83%)	92 (85%)	87 (83%)
Left‐sided	11 (10%)	7 (6%)	10 (10%)
Codominant	7 (7%)	9 (8%)	8 (7%)
Stenosis severity			
No stenosis	46 (43%)	31 (29%)	29 (28%)
< 50%	28 (26%)	41 (38%)	40 (38%)
≥ 50%	34 (31%)	36 (33%)	36 (34%)
Myocardial bridge	12 (11%)	21 (19%)	22 (21%)

### 3.2 Objective image quality

**FIGURE 3:**
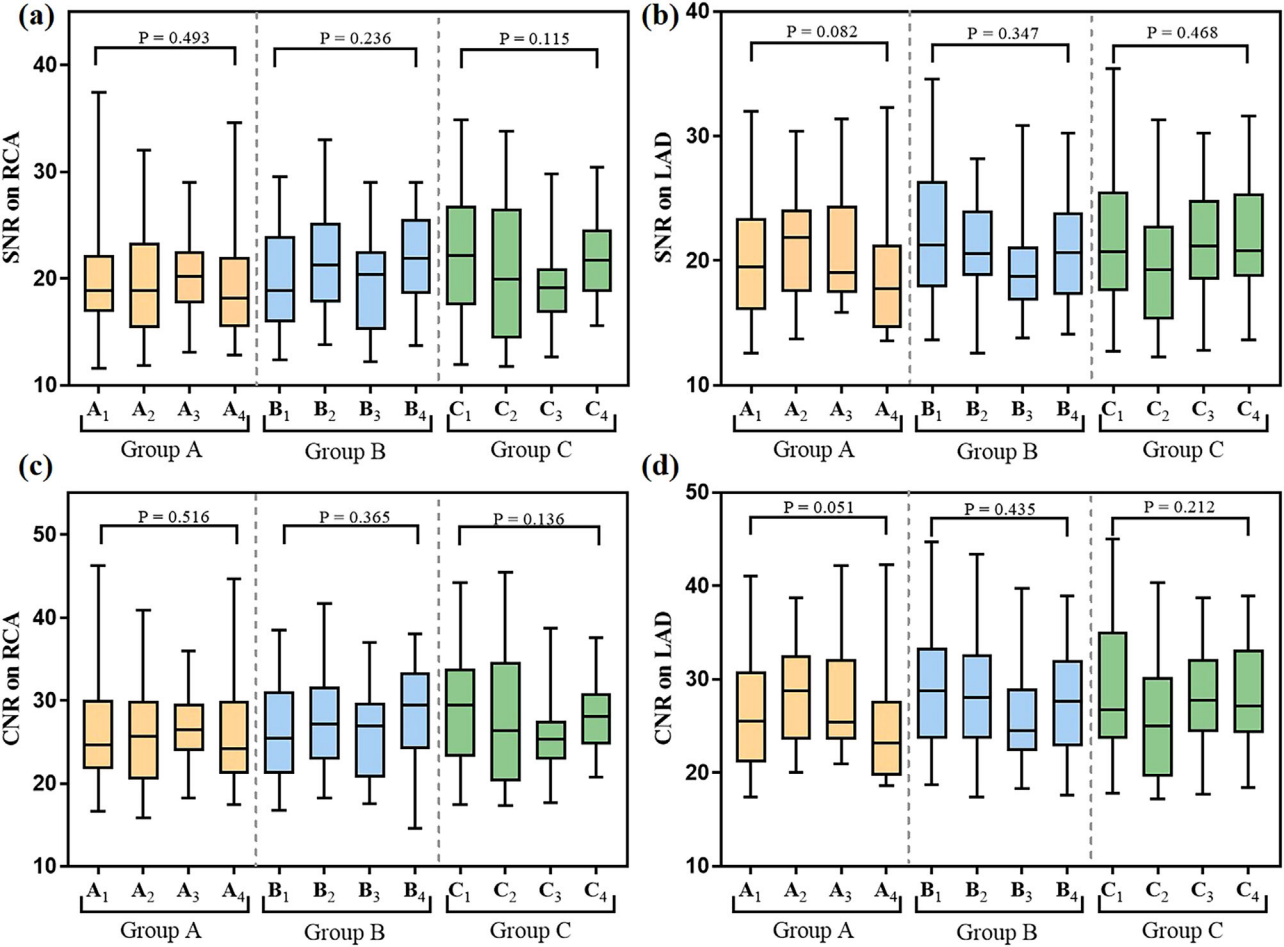
Comparison of objective image quality. SNRs, signal‐to‐noise ratios; CNRs, contrast‐to‐noise ratios; LAD, left anterior descending; RCA, right coronary artery.

The results of objective image quality scores are shown in Figure 3. For each weight group and for each artery under evaluation, no significant differences were found in SNR or CNR between the subgroups (all *p* > 0.05). The fact that such objective image quality metrics were not sensitive to the variation of both radiation and contrast medium dose parameters suggests additional factors must be taken into account for optimizing the scanning protocol. Although subgroups A_3_ and B_4_ shared the same setting, and so did subgroups B_3_ and C_4_, no significant difference was noticed: A_3_ versus B_4_, *p* = 0.347 for SNR on RCA, *p* = 0.352 for CNR on RCA, *p* = 0.993 for SNR on LAD, and *p* = 0.933 for CNR on LAD; B_3_ versus C_4_, *p* = 0.051 for SNR on RCA, *p* = 0.108 for CNR on RCA, *p* = 0.145 for SNR on LAD, and *p* = 0.121 for CNR on LAD. In particular, the same tube potential (80 kVp) was used for all these four subgroups, suggesting that the feasibility of applying a reduced kVp may not be necessarily unique to low‐weight patients.

### 3.3 Subjective image quality

Table 3 listed the subjective image quality scores, which were evaluated on a total of 3848 coronary segments in 321 cases. The average score for every subgroup was ≥3, in keeping with the study design that the variation of settings was not meant to substantially damage the image quality routinely already obtained and considered acceptable for diagnosis. The inter‐observer agreement was excellent for each case, with Kappa > 0.80. Subgroups B_2_ and A_1_ were set the same parameter settings, the mean scores of subgroups B_2_ and A_1_ were similar (3.84 ± 0.36 vs. 3.83 ± 0.44). In addition, subgroups B_4_ and A_3_ with the same parameter setting, the mean scores were also close (3.85 ± 0.37 vs. 3.84 ± 0.36). Similarly, subgroups B_1_ and C_2_, subgroups C_4_ and B_3_ with the same parameter setting were close in mean scores, respectively. This suggested that there was no significant difference in the subjective score of body weight at 55−85 kg with the same parameter setting. Score 1 was only received on two patients of group A, one patient on coronary 8th segment and one patient on coronary 13th segment, where the distal coronary arteries were exceptionally small and not yet fulfilled by the contrast medium.

**TABLE 3:** Subjective image quality scores for each subgroup.

Scores	1	2	3	4	Mean ± SD	Kappa
Group A 55−65 kg						
Subgroup A_1_	1 (0.3%)	4 (1.4%)	39 (13.3%)	250 (85.0%)	3.83 ± 0.44	0.82
Subgroup A_2_	0	2 (0.7%)	39 (13.7%)	243 (85.6%)	3.86 ± 0.36	0.88
Subgroup A_3_	0	1 (0.3%)	61 (16.5%)	306 (83.2%)	3.84 ± 0.36	0.92
Subgroup A_4_	1 (0.3%)	14 (4.6%)	82 (27.1%)	206 (68.0%)	3.65 ± 0.55	0.88
Group B 66−75 kg						
Subgroup B_1_	0	3 (0.8%)	28 (7.7%)	334 (91.5%)	3.89 ± 0.33	0.98
Subgroup B_2_	0	0	57 (17.8%)	264 (82.2%)	3.84 ± 0.36	0.87
Subgroup B_3_	0	1 (0.3%)	49 (15.6%)	264 (84.1%)	3.85 ± 0.36	0.91
Subgroup B_4_	0	3 (1.0%)	34 (11.6%)	245 (87.4%)	3.85 ± 0.37	0.84
Group C 76−85 kg						
Subgroup C_1_	0	1 (0.3%)	53 (14.6%)	309 (85.1%)	3.83 ± 0.37	0.98
Subgroup C_2_	0	3 (1.1%)	58 (20.7%)	219 (78.2%)	3.80 ± 0.41	0.82
Subgroup C_3_	0	1 (0.3%)	38 (9.7%)	352 (90.0%)	3.91 ± 0.30	0.91
Subgroup C_4_	0	2 (0.7%)	69 (25.4%)	201 (73.9%)	3.76 ± 0.42	0.86

### 3.4 Determination of the optimal setting

**FIGURE 4:**
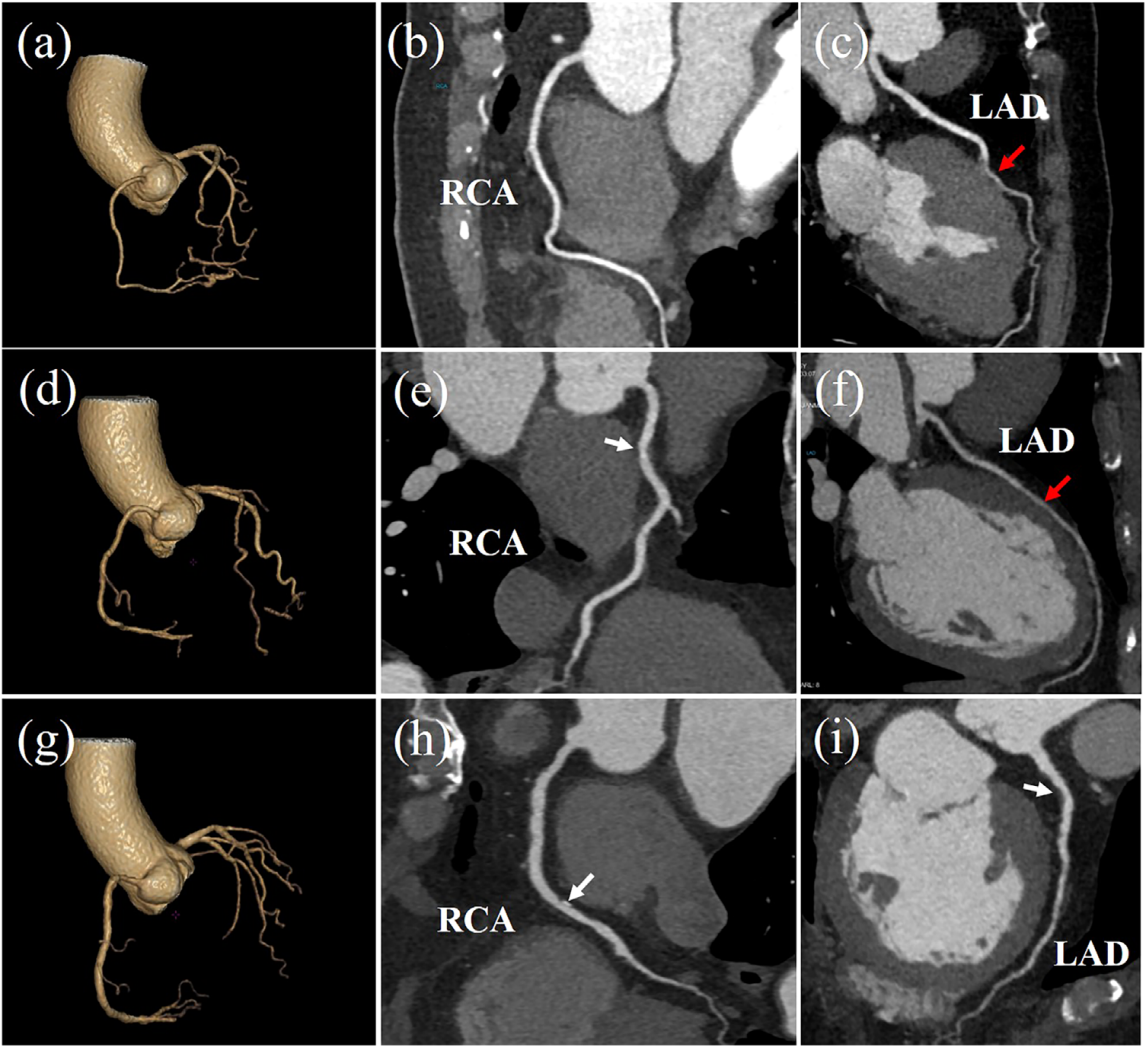
(a‐c) VR and CPR images of a 73‐year woman (weight, 60 kg; BMI, 22.58 kg/m^2^) in subgroup A_3_ (80 kVp, 150 mAs, 1.0 gI/s) diagnosed with myocardial bridge on LAD (red arrow). (d‐f) VR and CPR images of a 56‐year man (weight, 66 kg; BMI, 22.83 kg/m^2^) in subgroup B_4_ (80 kVp, 150 mAs, 1.0 gI/s) diagnosed with soft plaques and stenosis < 50% on RCA (write arrow) and myocardial bridge on LAD (red arrow). (g‐i) VR and CPR images of a 70‐year man (weight, 85 kg; BMI, 29.4 kg/m^2^) in subgroup C_3_ (100 kVp, 170 mAs, 1.5 gI/s) diagnosed with calcified plaque and stenosis = 50% on RCA and soft plaques and stenosis = 30% on LAD (write arrows). BMI, body mass index; CPR, curved planar reconstruction; VR, volume rendering.

All other subgroups of protocols SNR and CNR measurement have no difference in image quality from conventional scanning protocols (Subgroups A_1_, B_1_, and C_1_), meeting the requirements of image contrast. Based on the results above, the search for optimal protocol was determined by the subjective image quality in combination with the applied radiation dose. The subjective image quality was focused on the percentage of excellent images (score = 4). For group A, subgroups A_1_ (85.0%), A_2_ (85.6%), and A_3_ (83.2%) were similar in the percentage of score 4; however, subgroup A_3_ (80 kVp, 150 mAs, 1.0 gI/s) was considered a superior choice since the applied radiation dose was lower. For group B, subgroups B_1_ (91.5%) and B_4_ (87.4%) were relatively close in the percentage of score 4. But the applied radiation dose in subgroup B_1_ was found 161% higher than subgroup B_4_, therefore, subgroup B_4_ (80 kVp, 150 mAs, 1.0 gI/s) was the optimal scanning scheme for group B. For group C, the setting used in subgroup C_3_ (90.0%) was determined optimal (100 kVp, 170 mAs, 1.5 gI/s), given that the percentage of score 4 was highest. Figure 4 shows three cases of excellent image quality in the subgroups A_3_, B_4_, and C_3_, respectively.

## 4 DISCUSSION

In this study, we refined the currently used weight grouped protocol for CCTA in terms of the radiation and contrast medium dose through clinical evaluation. Given that no significant difference was found in objective image quality between subgroups, the optimal setting for each weight group was determined by the subjective image quality in combination with the applied radiation dose, where the subjective image quality was compared in terms of the percentage of cases being graded excellent, that is, receiving a score of 4. The detailed and comprehensive optimal scanning protocol was obtained by using the tentative approach in each body weight group, which provided diagnostically sufficient image quality for clinical diagnosis while reducing radiation dose and iodine intake.

The patients < 55  or > 85 kg was additionally carried out with a small number of cases. Two scanning protocols, one routine protocol (*n* = 10, 100 kVp, 100 mAs, 1.1 gI/s) and one “reduction” protocol (*n* = 8, 70 kVp, 220 mAs, 0.8 gI/s), were used on patients of 50−54 kg. Results showed that the objective and subjective image quality of the “reduction” protocol were not significantly different from that of the routine protocol (all *p* > 0.05). The “reduction” protocol (50−54 kg patients) with the same parameters as subgroup A_4_ (55−65 kg patients) showed no significant difference between objective or subjective scores (all *p* > 0.05). Similarly, patients with 86−90 kg were also used with one routine protocol (*n* = 9, 100 kVp, 200 mAs, 1.5 gI/s) and one “reduction” protocol (*n* = 8, 100 kVp, 170 mAs, 1.5 gI/s), where the objective and subjective image quality were not significantly different between two protocols (all *p* > 0.05). The “reduction” protocol (86−90 kg patients) was the same parameters with subgroup C_3_ (76−85 kg patients), but there was no significant difference in objective and subjective image quality (all *p* > 0.05). Although the number of patients of 50−54 kg and 85−90 kg was too small to be statistically sufficient, the results provided a reference for later attempts to optimize the dose settings for lean or obese patients.

In this study, the applied radiation dose was represented by the planned volume CT dose index (CTDI_vol_), instead of the dose integral that is received by the patient from the entire examination, such as in the dose‐length product (DLP) or effective dose (ED). While CTDI_vol_ characterizes the strength of radiation that is delivered by the scanner, the dose integral also depends on the longitudinal collimation and the length of the scan, which reflect the heart size and the number of acquired temporal phases, respectively, for each individual patient. In context of dose optimization as discussed in the present investigation, CTDI_vol_ was obviously the correct choice, providing the most essential, unbiased information on radiation dose.

Body mass index (BMI) has been used in prior studies as the grouping factor for patients. Yuan et al. explored the feasibility of low tube voltage and IDR determined by patients’ BMI in CCTA.^15^ The protocol of 100 kVp and 1.3 gI/s was used with BMI on 18.5−24.9 kg/m^2^, and the patients of BMI ≥25 kg/m^2^ were used with a protocol of 120 kVp and 1.6 gI/s. Li et al. explored the use of 70 kVp tube voltage in patients with BMI less than 26 kg/m^2^.^21^ As shown in Table 2, the difference of BMI for subjects enrolled in the present study was evident between three groups but numerically rather small. As such, body weight might be better suited for implementing a patient‐specific protocol for CCTA in practice, especially given the fact the variation of BMI for patients should not be large when those with extreme body characteristics are considered separately.

The present study had several limitations. First, the left circumflex artery was not considered in the objective image quality evaluation since the individual difference on circumflex artery is naturally quite large, which might complicate the evaluation of image quality. Second, while focused on image quality, the coronary artery stenosis found on CCTA images was not used, either as an extra grouping factor for image quality analysis or as a task for detectability test. This was in part due to the lack of gold standard of the diagnostic results. Third, this study considered only the hybrid iterative reconstruction that has been widely available with current commercial CT systems. It is of interest to investigate the same topic with emerging CT algorithms, such as deep‐learning based reconstruction, where the findings might be surprisingly different. Fourth, the automated exposure control (AEC) technique was not considered in this study, which would also be a reasonable starting point for optimization and worth comparing with the obtained parameter settings. Finally, the CCTA protocol refined in this study was only one scanner‐specific implementation. Care must be taken when translating the findings in this study to other CT models. The most determining factors are tube output, scanning geometry, and the image reconstruction method. Direct translation or scaling without a separate investigation is hardly reliable, even for those with similar wide detectors allowing volumetric CCTA in axial mode. However, the demonstrated investigative strategy for determining the optimal setting should be directly applicable to other scenarios, which is more essential than the exact value of the parameters.

## 5 CONCLUSION

It is feasible to refine the currently used, weight‐grouped protocol for CCTA in terms of radiation and contrast medium dose, by use of an optimization strategy where the balance between dose and image quality can be improved in a routine clinical setting. Apart from the optimal protocol as found for each of the weight group, the strategy is directly extensible to more detailed categorization of patient characteristics.

## AUTHOR CONTRIBUTION

Conceptualization and study design: Huishu Yuan and Guozhi Zhang; Data collection, analysis, and interpretation: Yan Zhang, Ying Wang, Jing Li, and Aihui Di; Manuscript preparation: Yan Zhang, Jing Li, and Guozhi Zhang; All authors contributed to the work and approved the submitted version.

## CONFLICT OF INTEREST STATEMENT

The authors report no conflicts of interest.
